# Supplementary material for: Effect of the molecular weight of water-soluble chitosan on its fat-/cholesterol-binding capacities and inhibitory activities to pancreatic lipase
Source: PeerJ. 2017 May 3;5:e3279. doi: 10.7717/peerj.3279 (PMC5419207; doi:10.7717/peerj.3279)
Supplement: Data S1 [file peerj-05-3279-s001.zip › raw data/raw data of Fig. 1ú1⁄44ú1⁄45ú1⁄49ú1⁄410ú1⁄411.docx]

**Raw data of Fig.1 The effect of reaction time on degradation of chitosan under the conditions of microwave irradiation (70 ◦C, 600W).**

| Reaction time(min) | 18 | 22 | 26 | 29 | 32 | 60 | 70 |
| --- | --- | --- | --- | --- | --- | --- | --- |
| Mw(10^3^kDa) | 7 | 7 | 6 | 5 | 3 | 1 | 0.8 |

**Raw data of Fig.4** **Fat-binding capacities of WSC, CTS and cellulose.**

|  | | the weight of beaker  (g, before the experiment) | the weight of beaker and bound oil  (g, after the experiment) |
| --- | --- | --- | --- |
| WSC1 | 1 | 112.21 | 113.32 |
|  | 2 | 105.04 | 106.21 |
|  | 3 | 109.06 | 110.26 |
| WSC2 | 1 | 107.28 | 107.94 |
|  | 2 | 109.63 | 11.3 |
|  | 3 | 108.23 | 108.89 |
| WSC3 | 1 | 108.03 | 108.85 |
|  | 2 | 104.63 | 105.55 |
|  | 3 | 196.34 | 107.21 |
| WSC4 | 1 | 104.25 | 105.76 |
|  | 2 | 108.29 | 109.78 |
|  | 3 | 109.76 | 111.26 |
| WSC5 | 1 | 104.85 | 106.09 |
|  | 2 | 106.39 | 107.52 |
|  | 3 | 109.28 | 110.47 |
| CTS0 | 1 | 111.75 | 111.93 |
|  | 2 | 105.09 | 105.25 |
|  | 3 | 107.7 | 107.89 |
| cellulose | 1 | 111.94 | 113.38 |
|  | 2 | 106.04 | 107.5 |
|  | 3 | 106.10 | 107.68 |
| blank | 1 | 110.4 | 111.94 |
|  | 2 | 110.3 | 111.78 |
|  | 3 | 110 | 111.52 |

**Raw data of Fig.5 Cholesterol- binding capacities of WSC, CTS and cellulose.**

|  | | A_510nm_ |
| --- | --- | --- |
| WSC1 | 1 | 0.068 |
|  | 2 | 0.072 |
|  | 3 | 0.069 |
| WSC2 | 1 | 0.063 |
|  | 2 | 0.064 |
|  | 3 | 0.065 |
| WSC3 | 1 | 0.066 |
|  | 2 | 0.064 |
|  | 3 | 0.064 |
| WSC4 | 1 | 0.075 |
|  | 2 | 0.076 |
|  | 3 | 0.078 |
| WSC5 | 1 | 0.079 |
|  | 2 | 0.077 |
|  | 3 | 0.08 |
| CTS0 | 1 | 0.086 |
|  | 2 | 0.081 |
|  | 3 | 0.086 |
| cellulose | 1 | 0.13 |
|  | 2 | 0.133 |
|  | 3 | 0.132 |
| blank | 1 | 0.142 |
|  | 2 | 0.134 |
|  | 3 | 0.140 |

**Raw data of Fig.9A Effect of temperature on lipase activity.**

|  | | A_405nm_ |
| --- | --- | --- |
| 30℃ | 1 | 0.446 |
|  | 2 | 0.456 |
|  | 3 | 0.438 |
| 33℃ | 1 | 0.464 |
|  | 2 | 0.47 |
|  | 3 | 0.47 |
| 37℃ | 1 | 0.474 |
|  | 2 | 0.476 |
|  | 3 | 0.476 |
| 40℃ | 1 | 0.364 |
|  | 2 | 0.4 |
|  | 3 | 0.408 |
| 45℃ | 1 | 0.326 |
|  | 2 | 0.336 |
|  | 3 | 0.33 |
| blank | 1 | 0.098 |
|  | 2 | 0.102 |
|  | 3 | 0.104 |

**Raw data of Fig.9B Effect of pH on lipase activity**

|  | | A_405nm_ |
| --- | --- | --- |
| pH=6.0 | 1 | 0.434 |
|  | 2 | 0.452 |
|  | 3 | 0.46 |
| pH=7.0 | 1 | 1.276 |
|  | 2 | 1.294 |
|  | 3 | 1.284 |
| pH=8.0 | 1 | 1.75 |
|  | 2 | 1.83 |
|  | 3 | 1.938 |
| pH=9.0 | 1 | 0.17 |
|  | 2 | 0.172 |
|  | 3 | 0.178 |
| pH=10.0 | 1 | 0.156 |
|  | 2 | 0.136 |
|  | 3 | 0.138 |
| blank | 1 | 0.106 |
|  | 2 | 0.114 |
|  | 3 | 0.112 |

**Raw data of Fig.9C, 10 Effect of substrate concentration on reaction speed**

|  | | A_405nm_ |
| --- | --- | --- |
| 2×10^-4^mol/L | 1 | 0.156 |
|  | 2 | 0.156 |
|  | 3 | 0.15 |
| 4×10^-4^mol/L | 1 | 0.19 |
|  | 2 | 0.196 |
|  | 3 | 0.198 |
| 6×10^-4^mol/L | 1 | 0.242 |
|  | 2 | 0.246 |
|  | 3 | 0.252 |
| 8×10^-4^mol/L | 1 | 0.28 |
|  | 2 | 0.282 |
|  | 3 | 0.286 |
| 10×10^-4^mol/L | 1 | 0.344 |
|  | 2 | 0.35 |
|  | 3 | 0.352 |
| 12×10^-4^mol/L | 1 | 0.358 |
|  | 2 | 0.364 |
|  | 3 | 0.366 |
| 14×10^-4^mol/L | 1 | 0.384 |
|  | 2 | 0.388 |
|  | 3 | 0.388 |
| blank | 1 | 0.108 |
|  | 2 | 0.112 |
|  | 3 | 0.122 |

**Raw data of Fig.9D Effect of enzyme concentration on reaction speed**

|  | | A_405nm_ |
| --- | --- | --- |
| 0.1mg/mL | 1 | 0.292 |
|  | 2 | 0.292 |
|  | 3 | 0.294 |
| 0.2mg/mL | 1 | 0.302 |
|  | 2 | 0.304 |
|  | 3 | 0.304 |
| 0.3mg/mL | 1 | 0.308 |
|  | 2 | 0.308 |
|  | 3 | 0.308 |
| 0.4mg/mL | 1 | 0.312 |
|  | 2 | 0.316 |
|  | 3 | 0.32 |
| 0.5mg/mL | 1 | 0.32 |
|  | 2 | 0.318 |
|  | 3 | 0.328 |
| 0.6mg/mL | 1 | 0.318 |
|  | 2 | 0.322 |
|  | 3 | 0.322 |
| 0.7mg/mL | 1 | 0.324 |
|  | 2 | 0.326 |
|  | 3 | 0.328 |
| blank | 1 | 0.272 |
|  | 2 | 0.282 |
|  | 3 | 0.288 |

**Raw data of Fig.9E Effect of adding order of substrate, enzyme, inhibitor on inhibition rate**

|  | | A_405nm_ |
| --- | --- | --- |
| order1 | 1 | 0.296 |
|  | 2 | 0.302 |
|  | 3 | 0.304 |
| order2 | 1 | 0.286 |
|  | 2 | 0.294 |
|  | 3 | 0.298 |
| order3 | 1 | 0.28 |
|  | 2 | 0.28 |
|  | 3 | 0.274 |
| blank | 1 | 0.1 |
|  | 2 | 0.102 |
|  | 3 | 0.106 |

**Raw data of Fig.11 Inhibition rate of pancreatic lipase by WSC, CTS and orlistat**

|  | | WSC1 | WSC2 | WSC3 | WSC4 | WSC5 | CTS0 | orlistat |
| --- | --- | --- | --- | --- | --- | --- | --- | --- |
| 1μg/mL | 1 | 0.104 | 0.108 | 0.116 | 0 | 0.118 | 0.118 | 4.438 |
|  | 2 | 0.106 | 0.108 | 0.118 | 0.24 | 0.114 | 0.118 | 4.35 |
|  | 3 | 0.112 | 0.114 | 0.116 | 0.18 | 0.12 | 0.12 | 4.464 |
| 10μg/mL | 1 | 0.104 | 0.11 | 0.118 | 0.12 | 0.12 | 0.118 | 4.308 |
|  | 2 | 0.106 | 0.11 | 0.122 | 0.118 | 0.12 | 0.122 | 4.28 |
|  | 3 | 0.11 | 0.114 | 0.122 | 0.124 | 0.118 | 0.122 | 4.192 |
| 25μg/mL | 1 | 0.1 | 0.114 | 0.114 | 0.112 | 0.118 | 0.118 | 4.204 |
|  | 2 | 0.11 | 0.114 | 0.122 | 0.122 | 0.122 | 0.12 | 4.174 |
|  | 3 | 0.108 | 0.112 | 0.124 | 0.124 | 0.13 | 0.126 | 4.186 |
| 50μg/mL | 1 | 0.108 | 0.114 | 0.122 | 0.124 | 0.12 | 0.126 | 4.022 |
|  | 2 | 0.112 | 0.114 | 0.126 | 0.126 | 0.128 | 0.126 | 4.076 |
|  | 3 | 0.112 | 0.116 | 0.126 | 0.126 | 0.122 | 0.128 | 4.026 |
| 100μg/mL | 1 | 0.108 | 0.114 | 0.124 | 0.12 | 0.12 | 0.124 | 4.024 |
|  | 2 | 0.114 | 0.116 | 0.126 | 0.126 | 0.122 | 0.122 | 4.062 |
|  | 3 | 0.114 | 0.118 | 0.128 | 0.126 | 0.122 | 0.12 | 4.072 |
| Blank control of WSC1-5 | 1 | 0.102 |  |  |  |  |  |  |
|  | 2 | 0.108 |  |  |  |  |  |  |
|  | 3 | 0.108 |  |  |  |  |  |  |
| Blank control of orlistat | 1 | 4.282 |  |  |  |  |  |  |
|  | 2 | 3.376 |  |  |  |  |  |  |
|  | 3 | 4.43 |  |  |  |  |  |  |
| positive control | 1 | 4.484 |  |  |  |  |  |  |
|  | 2 | 4.438 |  |  |  |  |  |  |
|  | 3 | 4.456 |  |  |  |  |  |  |
| negative control | 1 | 0.12 |  |  |  |  |  |  |
|  | 2 | 0.12 |  |  |  |  |  |  |
|  | 3 | 0.12 |  |  |  |  |  |  |
